# Tailored Web-Based Interventions for Pain: Systematic Review and Meta-Analysis

**DOI:** 10.2196/jmir.8826

**Published:** 2017-11-10

**Authors:** Geraldine Martorella, Madalina Boitor, Melanie Berube, Suzanne Fredericks, Sylvie Le May, Céline Gélinas

**Affiliations:** ^1^ College of Nursing Florida State University Tallahassee, FL United States; ^2^ Tallahassee Memorial Hospital Center for Research and Evidence-Based Practice Tallahassee, FL United States; ^3^ Quebec Nursing Intervention Research Network (RRISIQ) Montreal, QC Canada; ^4^ Ingram School of Nursing McGill University Montreal, QC Canada; ^5^ Department of Trauma Centre Integré Universitaire du Nord de l'Île de Montréal Hôpital du Sacré-Cœur de Montréal Montreal, QC Canada; ^6^ Department of Nursing Centre Integré Universitaire du Nord de l'Île de Montréal Hôpital du Sacré-Cœur de Montréal Montreal, QC Canada; ^7^ Daphne Cockwell School of Nursing Ryerson University Toronto, ON Canada; ^8^ Faculté des sciences infirmières Université de Montréal Montreal, QC Canada; ^9^ Centre de recherche Centre hospitalier universitaire Ste Justine Montreal, QC Canada; ^10^ Center for Nursing Research Jewish General Hospital McGill University Montreal, QC Canada; ^11^ Alan Edwards Centre for Research on Pain McGill University Montreal, QC Canada; ^12^ Lady Davis Institute Jewish General Hospital McGill University Montreal, QC Canada

**Keywords:** Web-based intervention, tailored intervention, pain management, chronic pain, acute pain, review, systematic review, meta-analysis

## Abstract

**Background:**

Efforts have multiplied in the past decade to underline the importance of pain management. For both acute and chronic pain management, various barriers generate considerable treatment accessibility issues, thereby providing an opportunity for alternative intervention formats to be implemented. Several systematic reviews on Web-based interventions with a large emphasis on chronic pain and cognitive behavioral therapy have been recently conducted to explore the influence of these interventions on pain management However, to our knowledge, the specific contribution of tailored Web-based interventions for pain management has not been described and their effect on pain has not been evaluated.

**Objective:**

The primary aim of this systematic review was to answer the following research question: What is the effect of tailored Web-based pain management interventions for adults on pain intensity compared with usual care, face-to-face interventions, and standardized Web-based interventions? A secondary aim was to examine the effects of these interventions on physical and psychological functions.

**Methods:**

We conducted a systematic review of articles published from January 2000 to December 2015. We used the DerSimonian-Laird random effects models with 95% confidence intervals to calculate effect estimates for all analyses. We calculated standardized mean differences from extracted means and standard deviations, as outcome variables were measured on different continuous scales. We evaluated 5 different outcomes: pain intensity (primary outcome), pain-related disability, anxiety, depression, and pain catastrophizing. We assessed effects according to 3 time intervals: short term (<1 month), medium term (1-6 months), and long term (6-12 months).

**Results:**

After full-text review, we excluded 31 articles, resulting in 17 eligible studies. Only 1 study concerned acute pain and was removed from the meta-analysis, resulting in 16 studies available for quantitative assessment. Compared with standard care or a waiting list, tailored Web-based intervention showed benefits immediately after, with small effect sizes (<0.40) for pain intensity (10 randomized controlled trials [RCTs], n=1310, *P*=.003) and pain-related disability (6 RCTs, n=953, *P*<.001). No other improvements were observed at follow-up in the medium and long terms. Compared with the active control group, no improvements were found for the primary outcome (pain intensity) or any of the outcomes except for a small effect size on pain catastrophizing (2 RCTs, n=333, *P*<.001) immediately after the intervention.

**Conclusions:**

Tailored Web-based interventions did not prove to be more efficacious than standardized Web-based interventions in terms of pain intensity, pain-related disability, anxiety, and depression. An interesting finding was that some efficacy was shown on pain catastrophizing compared with active control interventions. Considering the diversity of approaches used in tailored Web-based interventions for chronic pain management, their efficacy is yet to be explored. Moreover, their contribution to acute pain management is embryonic.

**Trial Registration:**

International prospective register of systematic reviews (PROSPERO): CRD42015027669; http://www.crd.york.ac.uk/PROSPERO/display_record.php?ID=CRD42015027669 (Archived by WebCite at http://www. webcitation.org/6uneWAuyR)

## Introduction

Efforts have been made in the past decade to underscore the importance of pain management and its status as a human right [1-3]. Acute pain is a widespread issue. Annually, 300 million surgeries are performed worldwide, with a third occurring in the United States, and have resulted in approximately 80% of patients reporting pain [4]. As well, 70% of emergency departments visits are related to acute pain [4]. Furthermore, it is estimated that approximately 100 million adults in the United States have chronic pain [3], with 25.3 million adults experiencing daily pain [5]. Although caseloads and wait times are difficult to estimate, particularly in the United States, it is generally recognized that treatment availability for chronic pain patients is scarce [6,7]. Significant barriers such as time, cost, and distance generate considerable treatment accessibility issues [3] and inhibit the improvement of pain management, thereby providing an opportunity for alternative formats to face-to-face interventions to be implemented [8-10].

Over the past decade, more Web-based interventions for pain management have been developed and, as opposed to non-Web-based interventions, they have been shown to positively influence health behaviors [11,12]. Many terms have been used interchangeably to qualify Web-based interventions that facilitate the implementation of self-management health-related interventions. Here the term Web-based interventions refers to Barak et al’s definition [13]:

...a primarily self-guided intervention program that is executed by means of a prescriptive online program operated through a website and used by consumers seeking health- and mental-health[-]related assistance. The intervention program itself attempts to create positive change and or improve/enhance knowledge, awareness, and understanding via the provision of sound health-related material and use of interactive Web-based components.

This definition is composed of three types of health-related interventions: educational, self-guided therapeutic, and human-supported therapeutic interventions [13].

Several systematic reviews on Web-based interventions for pain, with a large emphasis on chronic pain issues and cognitive behavioral therapy (CBT) interventions, have been conducted to explore their influence on pain management [14-19]. Overall, their authors concluded that results were promising in terms of pain reduction, and improvement of functional and emotional well-being. They also underlined that it is still unknown as to which type of patients, according to sociodemographic and clinical characteristics, would benefit most from a Web-based approach [14,15]. A small positive effect was found on pain reduction, but results remain inconclusive considering the small sample sizes for several studies, high dropout rates, and heterogeneity related to assessment tools used and times of measurement selected, type of pain-related diseases, and interventions (eg, content, format, dose), but also the lack of diversity in patients (eg, mainly women, white, and college educated) [10,14,15,17,19]. Nonetheless, none of the systematic reviews looking at Web-based interventions for pain management specifically addressed the contribution of tailoring ingredients.

Experts in health behavior change have shown that conveying health information without considering individual differences may inhibit behavior change [20-26]. Tailoring strategies respond to this concern and computed algorithms can facilitate the implementation of this approach in terms of both accessibility and level of refinement. Tailoring is defined as a process for creating individualized communications using personal data related to health outcomes in order to meet individual needs [21,23,25,27,28]. Three mechanisms have been highlighted [21,29]: (1) personalization, which helps increase the perceived meaningfulness of the message by creating the impression that the message was designed specifically for the individual [21]; (2) feedback, which directs the attention of the individual to their own characteristics or behaviors that they need to address, improve, or change [21]; and (3) adaptation or content matching, which refers to creating content packages that are pertinent to an individual and are selected based on known determinants of the targeted behavior [21]. Clinically relevant results, such as adopting a healthy lifestyle or adhering to medication, and statistically significant effect sizes of tailored Web-based interventions have been recognized for health behavior change among diverse populations facing chronic disease [20,29,30]. However, the contribution of tailored Web-based interventions for pain management has not been described, and their specific effect on pain has not been evaluated.

Therefore, this systematic review aimed to answer the following research question: What is the effect of tailored Web-based pain management interventions for adults on pain intensity compared with usual care, face-to-face interventions, and standardized Web-based interventions? We also examined secondary outcomes related to the effects of these interventions on physical and psychological functions.

## Methods

This systematic review protocol has been developed based on Preferred Reporting Items for Systematic Reviews and Meta-Analyses guidelines for reporting systematic reviews evaluating health care interventions [31-33]. The detailed protocol was published [34] and registered with the international prospective register of systematic reviews (no CRD42015027669).

### Inclusion Criteria

We include solely randomized controlled trials (RCTs) in this systematic review. Other inclusion criteria were studies with patients 18 years of age or older and experiencing any type of pain (acute, ie, <3 months; chronic, ie, 3-6 months and beyond [35]). We selected studies involving Web-based interventions for pain management including at least one of the three tailoring strategies (personalization, feedback, or adaptation) [21,29]. Eligible comparators were (1) a passive control group (ie, participants receiving usual medical and nursing care or being on a pain clinic waitlist), and (2) active control group (eg, face-to-face educational or psychological intervention or Web-based standardized intervention) [14,15].

We selected outcomes according to pain clinical trials recommendations [36-38]. Pain intensity was a mandatory outcome for the study to be included in this systematic review. The timeline of outcomes included measures before and immediately after treatment and at follow-up. To reduce selection bias, if articles were published in languages other than English or French, we reviewed the English abstract to determine whether the study should be translated and included. This was the case for an article published in German identified as eligible [39] and translated by a member of the team (MaB).

### Search Strategy

We searched MEDLINE, Embase, CINAHL, PsycINFO, Web of Science, and the Cochrane Library for articles published from January 2000 to December 2015. Reviewing the reference lists of relevant articles and previous systematic reviews helped identify 9 additional articles. Moreover, an experienced research librarian used subject headings to avoid missing nonindexed concepts. Search terms were “pain,” “pain management,” “program,” “intervention,” “Internet,” “Internet-based,” “online,” “Web-based,” and “mobile OR mobile applications” [34].

### Screening and Selection of Studies

Eligibility was assessed independently in an unblinded standardized manner by 2 team members (GM, CG) and results were then compared. Titles and abstracts were screened. If a trial was potentially eligible, the full text was reviewed. The 2 reviewers are researchers in the field of pain with a clinical background in nursing. Disagreements between reviewers at the full-text level were discussed until consensus was reached.

### Data Extraction and Management

Data were extracted independently by 2 teams of 2 reviewers (GM and MaB; CG and MeB) composed of 1 doctoral student and 1 researcher using the software DistillerSR v2 (Evidence Partners Inc). We developed the data extraction form we used based on the Cochrane Consumers and Communication Group’s data extraction template [40], pilot tested it with 5 articles and refined it accordingly (eg, number of comparator arms, time points for postintervention assessments). Disagreements between reviewers were resolved by discussion between the 2 teams until 100% agreement was reached. We requested missing data such as means and standard deviations regarding the outcome variables from authors. Extracted data included sample size, sample demographics, dropout rate, number and type of study groups, type and location of pain, inclusion and exclusion criteria, study setting, type of Web-based intervention (ie, setting, mode, dose, contact with therapist, hybrid format), tailoring strategy (ie, personalization, feedback, adaptation), comparator (ie, passive control group vs active control group), type of pain intensity measure, pain-related disability and psychological well-being outcomes, and times of measurement.

### Data Assessment and Synthesis

Risk of bias for the 17 selected studies was assessed by the 2 teams of reviewers using the Cochrane Collaboration tool [41-43], and any discrepancies between reviewers were discussed between the 2 teams. The report of the risk-of-bias assessment is presented in the Results section.

We used Review Manager (RevMan 5.3; Cochrane Collaboration) software [44] for statistical analysis. We used the DerSimonian-Laird random effects models with 95% confidence intervals to calculate effect estimates for all analyses. We calculated standardized mean differences (SMDs) from extracted means and standard deviations, as outcome variables were measured on different continuous scales. We planned subgroup analyses by the comparator (ie, passive or active control) and type of pain (ie, acute or chronic). The primary outcome was pain intensity measured using a self-report measure such as the numeric rating scale (NRS) (eg,) or visual analog scale (VAS) (eg, 0-10 cm or 0-100 mm). We converted scores reported on a 0-100 mm VAS to 0-10 NRS scores for the purpose of data analysis [45]. If authors provided data for least, average, and worst pain intensity as measured by the Brief Pain Inventory, we used average scores for data analysis. Secondary outcomes, if available, were pain-related disability (eg, Brief Pain Inventory; Roland Morris Disability Questionnaire), and psychological well-being (eg, Hospital Anxiety and Depression Scale; Pain Catastrophizing Scale). In an effort to decrease the heterogeneity found in pain-related disability and psychological well-being measures, we included tools measuring the same construct to calculate SMDs. Given the variability in follow-up assessments across the included studies, we report outcomes according to 3 different time intervals: (1) short-term effect: immediately after or within a month after intervention, (2) medium-term effect: up to 6 months after completion of the intervention, (3) long-term effects: over 6 months after completion of the intervention. We evaluated between-study variability using the method proposed by Higgins et al [42]. We considered an I^2^ statistic above 50% to indicate high heterogeneity, values between 25% and 50% to indicate moderate heterogeneity, and those below 25% to indicate low heterogeneity.

## Results

**Figure 1:**
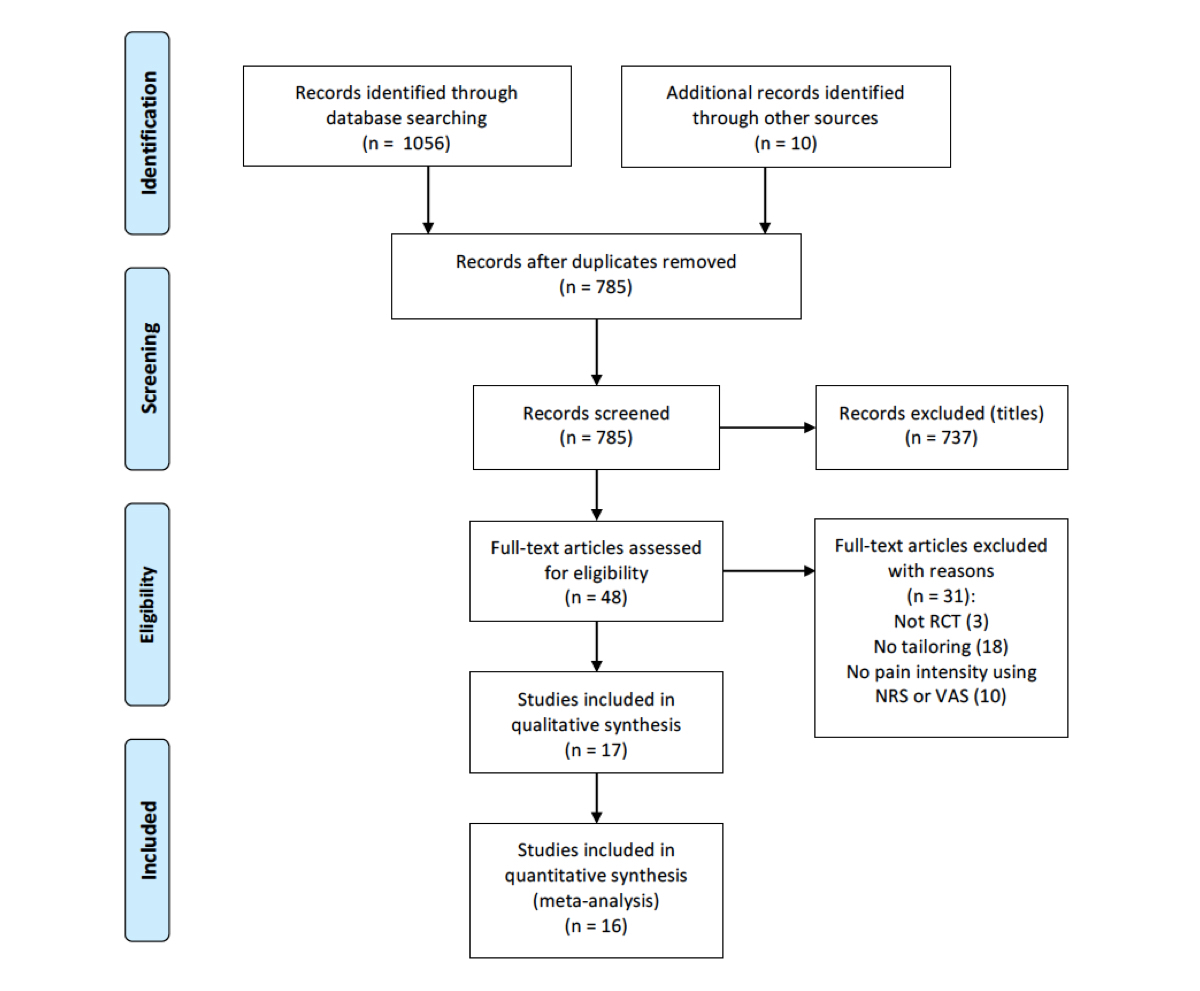
Preferred Reporting Items for Systematic Reviews and Meta-Analyses (PRISMA) flowchart. NRS: numeric rating scale; RCT: randomized controlled trial; VAS: visual analog scale.

After a full-text review of 48 articles, we excluded 31 for the following reasons: 3 studies were not RCTs, 18 studies did not involve 1 of the tailoring mechanisms, and 10 studies did not measure pain intensity using a VAS or NRS or a pain index calculated with scores obtained on an NRS over a period of time. We included 17 studies in the qualitative synthesis and 16 in the meta-analysis (see Figure 1). Of note, the effects of 1 intervention were described in 2 articles: short- and medium-term effects [46] and long-term effects [47].

### Study Characteristics

As Table 1 presents, we included 17 studies, 16 of which were performed in the chronic pain context [39,46-61] and 1 in the acute pain context in the postcardiac surgery phase [62]. Concerning studies conducted in the chronic pain context (n=16), 6 included individuals with back pain [39,50-52,55,59], 3 included individuals with other specific pain sites (head as well as hips and knees) [48,49,61], 5 included individuals with multiple pain sites or widespread pain [46,47,53,54,58,60], and 2 included individuals with chronic disease(s) (ie, heart disease, lung disease, type 2 diabetes, mobility difficulty, chronic musculoskeletal pain, and depression) [56,57]. Considering that only 1 study was performed in the acute care context, we included only data from chronic pain studies in the meta-analysis.

**Table 1:** Characteristics of the 17 eligible studies.

First author, year, reference	Country	Sample size (n)	Lost to follow-up^a^ (%)	Age in years, mean (SD)	Female (%)	Type of pain	Pain location
Andersson, 2003 [48]	Sweden	44	45.5	40.3 (NR^b^)	81.7	Chronic	Headache
Bossen, 2013 [49]	Netherlands	199	15.6	62 (5.7)	64.8	Chronic	Hips, knees
Buhrman, 2004 [50]	Sweden	56	8.9	44.6 (10.4)	62.5	Chronic	Back
Carpenter, 2012 [51]	United States	141	7.1	42.5 (10.3)	83	Chronic	Low back
Chiauzzi, 2010 [52]	United States	209	11	46.1 (11.9)	67	Chronic	Back
Dear, 2013 [53]	Australia	63	4.8	49 (13)	86	Chronic	Multiple
Dear, 2015 [54]	Australia	490	14.1	50 (13)	80	Chronic	Multiple
Krein, 2013 [55]	United States	229	9.6	51.5 (NR)	12.5	Chronic	Back
Kristjánsdóttir, 2013 [46,47]	Norway	140	40	44.2 (NR)	100	Chronic	General
Leveille, 2009 [56]	United States	241	22.8	52 (12)	57	Chronic	Chronic disease related
Lorig, 2006 [57]	United States	958	18.2	57.5 (10.9)	71	Chronic	Chronic disease related
Lorig, 2008 [58]	United States	855	25	52 (11.6)	90	Chronic	Arthritis/ fibromyalgia
Martorella, 2012 [62]	Canada	60	13.3	64.6 (8.2)	21	Acute	Surgical site
Moessner, 2012 [59]	Germany	75	44	45.9 (NR)	57	Chronic	Back
Moessner, 2014 [39]	Germany	334	21.3	47.36 (9.89)	63.8	Chronic	Back
Shigaki, 2013 [60]	United States	108	13.9	49.8 (NR)	92.3	Chronic	General
Ström, 2000 [61]	Sweden	102	56	36.7 (NR)	68	Chronic	Head

^a^Rates are calculated based on the number of randomly assigned participants who completed a posttreatment questionnaire (time points may vary within studies).

^b^NR: not reported.

The total number of participants entering chronic pain trials was 4103 (mean 256.4 participants per study, SD 270.8, median 170, interquartile range 81.8-310.8). All studies described the total number of participants providing data at the end of the interventions. The mean completion rate for studies that provided such data was 77.6%, with the proportion of completers ranging across studies from 44% to 95.2%. The mean age of participants entering the studies was 48.2 years (SD 6.3, range 36.7-62.0, median 48.2, interquartile range 44.3-51.9). The average proportion of female participants was 71%.

A total of 5 studies used usual care as the comparator arm [39,55,57-59], 6 used a waiting list [49-51,53,60,61], and 5 included active controls, in which participants received Web-based information, psychological support, or standardized CBT without in-person contact with a therapist [46-48,52,54,56]. All studies used 1 comparator arm, except for 1 study that used 3: standardized CBT and tailored psychological support with optional health professional contact; standardized CBT and tailored psychological support without health professional contact; and a waiting list [54]. The interventions included 5 to 18 sessions over a period of 3 weeks to 12 months, and most of them were provided weekly.

A total of 13 studies evaluated Web-based interventions using a CBT or behavioral approach [39,46-54,57-60], which was combined with an additional approach (ie, education, relaxation, mindfulness therapy, and motivational and psychological support) in 9 studies [46-48,50-54,57,58]. Exercise, motivation, coaching, education, and relaxation approaches were used in the 3 trials that did not use CBT [55,56,61]. A total of 7 interventions were delivered with a hybrid mode of delivery precisely combining Internet and in-person contact with a health professional over the telephone or face-to-face [39,46-48,50,53,54,60]. Feedback (mediated or not) was used in every intervention. Content matching was used in half of the studies [39,46,47,49,52,55,57-59]. Personalization was difficult to assess given the lack of a detailed description of interventions and platforms in research articles, but also because it was embedded in the 2 other tailoring mechanisms. Table 2 summarizes the approaches and dosage of tailored Web-based interventions and their comparator.

**Table 2:** Description of tailored Web-based interventions and their comparator.

First author, year, reference	Web-based tailored intervention				Comparator	
	Approach	Format	Duration	Frequency	Approach	Type
Andersson, 2003 [48]	Relaxation + CBT^a^	Hybrid: telephone	6 weeks	1/week (6 sessions)	Same as intervention	Active (Web based)
Bossen, 2013 [49]	Behavioral graded activity	Hybrid: N/A^b^	9 weeks	1/week (9 sessions)	N/A	Waiting list
Buhrman, 2004 [50]	CBT + relaxation, exercise, and stretching	Hybrid: telephone	6 weeks	1/week (6 sessions)	N/A	Waiting list
Carpenter, 2012 [51]	CBT + relaxation, mindfulness	Hybrid: N/A	3 weeks	2/week (6 sessions)	N/A	Waiting list
Chiauzzi, 2010 [52]	CBT + motivational + educational (wellness, lifestyle)	Hybrid: N/A	4 weeks + 5 monthly boosters (6 months)	2/week (8 sessions)	Emailed back pain information guide	Active
Dear, 2013 [53]	CBT + educational (sleep hygiene)	Hybrid: telephone	8 weeks	Every 7-10 days (5 sessions)	N/A	Waiting list
Dear, 2015 [54]	CBT + psychological approach	Hybrid: telephone	8 weeks	Every 7-10 days (5 sessions)	Same as intervention	Active (Web based) and waitlist
Krein, 2013 [55]	Exercise, motivational	Hybrid: N/A	12 months	Weekly feedback, reminder (daily sessions)	Wearing a pedometer and reminders to upload data	Usual care
Kristjánsdóttir, 2013 [46,47]	CBT + ACT^c^ + mindfulness	Hybrid: face-to-face	4 weeks	5/week (20 sessions)	Information website	Active
Leveille, 2009 [56]	Coaching + educational (disease specific)	Hybrid: N/A	4 weeks	N/A	URL links provided to patients: home pages for the US Department of Health and Human Services and the Centers for Disease Control and Prevention	Active
Lorig, 2006 [57]	CBT + educational (nutrition, medication)	Hybrid: N/A	6 weeks	3/week (18 sessions)	N/A	Usual care
Lorig, 2008 [58]	CBT + educational (nutrition, medication)	Hybrid: N/A	6 weeks	3/week (18 sessions)	N/A	Usual care
Martorella, 2012 [62]	CBT	Hybrid: face-to-face	30 minutes + 2 boosters (5-10 minutes)	1 session before surgery	N/A	Usual care
Moessner, 2012 [59]	Behavioral	Hybrid: N/A	12-15 weeks	1/week (12-15 sessions)	N/A	Usual care
Moessner, 2012 [39]	CBT	Hybrid: face-to-face	12-15 weeks	1/week (12-15 sessions)	N/A	Usual care
Shigaki, 2013 [60]	CBT	Hybrid: telephone	10 weeks	1/week (10 sessions)	N/A	Waiting list
Ström, 2000 [61]	Relaxation	Hybrid: N/A	6 weeks	1/week (6 sessions)	N/A	Waiting list

^a^CBT: cognitive behavioral therapy.

^b^N/A: not applicable.

^c^ACT: acceptance and commitment therapy.

**Table 3:** Risk of bias within studies according to reviewers.

First author, year, reference	Random sequence generation	Allocation concealment	Blinding of participants and personnel	Blinding of outcome assessment	Incomplete outcome data	Selective reporting	Other bias
Andersson, 2003 [48]	Unclear	Unclear	Low	Low	High	Unclear	Low
Bossen, 2013 [49]	Low	Low	High	High	Low	Low	Low
Buhrman, 2004 [50]	Low	Unclear	High	High	Low	Low	Low
Carpenter, 2012 [51]	Low	Unclear	High	High	High	Low	Low
Chiauzzi, 2010 [52]	Low	Unclear	Unclear	Unclear	Low	Low	Low
Dear, 2013 [53]	Unclear	Unclear	High	High	Low	Low	Low
Dear, 2015 [54]	Low	Low	High	High	Low	Low	Low
Krein, 2013 [55]	Low	Low	High	High	Low	Low	Low
Kristjánsdóttir, 2013 [46,47]	Low	Low	High	High	Low	Low	Low
Leveille, 2009 [56]	Unclear	Low	High	High	High	Low	Low
Lorig, 2006 [57]	Unclear	Unclear	High	High	High	Low	Low
Lorig, 2008 [58]	Unclear	Low	High	High	High	Low	Low
Martorella, 2012 [62]	Low	Low	High	High	Low	Low	Low
Moessner, 2012 [59]	Unclear	Unclear	High	High	Low	Unclear	Unclear
Moessner, 2014 [39]	Unclear	Unclear	High	High	Low	Unclear	Unclear
Shigaki, 2013 [60]	Unclear	Unclear	High	High	High	Low	Unclear
Ström, 2000 [61]	Unclear	Unclear	High	High	High	Unclear	Low

### Risk-of-Bias Assessment

A total of 6 potential biases were evaluated according to the Cochrane Collaboration tool: selection, performance, detection, attrition, reporting, and other [42]. Regarding selection bias, randomized sequence generation presented an unclear risk for about half of the studies (n=9) [39,48,53,56-61] and a low risk of bias for the other half (n=8). Unclear risk was mainly related to insufficient information on the sequence generation. It was also the case for allocation concealment, which we judged to be unclear for 10 studies [39,48,50-53,57,59-61]. In terms of performance bias, all studies except for 2 (low and unclear risks) [48,52] presented a high risk in regard to blinding of participants, which was also the case for detection bias. These 2 biases are almost inevitable with this type of intervention, especially compared with a waitlist, as the group assignment is easy to guess for participants who are the actual outcome assessors. Indeed, most of the time, outcomes were measured through self-administered online questionnaires. The study by Andersson et al [48] presented a low risk because both groups received the same Web-based intervention and completed online questionnaires, with the only difference being a telephone contact added in the experimental group. Another study [51] used this strategy and provided the same intervention to both groups but not at the same time. The control group had to complete questionnaires before receiving the intervention, which ended up presenting a risk of bias. The risk in the study by Chiauzzi et al [52] remained unclear because of a lack of information. Blinding could have occurred even though the control group did not access a website but received a guide to consult. We found attrition risk of bias in 7 studies that presented a high risk regarding incomplete data [48,51,56-58,60,61]. The reasons for attrition were sometimes not explained, and information was lacking regarding the method for handling missing data. We judged selective reporting as low risk except in 4 studies (unclear) [39,59]. A total of 9 studies had registered protocols [46,47,49,53-56,58,60,62] and, for those without, we judged a low risk for selective reporting given the consistency between methods and results. Other risks of bias were low, except in 3 studies (unclear), in which some information was lacking regarding the methods and attrition. Table 3 summarizes the risk-of-bias assessment for each study. We constructed funnel plots for only 1 analysis due to the small number of studies included in each analysis (n<10) [63,64].

### Effects of Interventions

We included 16 studies in the quantitative analysis, having removed the single study on acute pain [62]. We conducted 2 main meta-analyses based on the type of comparator: tailored Web-based intervention versus standard care or waitlist control, and tailored Web-based intervention versus active control. For both meta-analyses, when possible, we analyzed outcomes at 3 different time points (ie, short, medium, and long term). Table 4 reports the tools used for outcome assessment and timeline per study. We included the study with 3 control groups (ie, optional contact, no contact with therapist, and standard care or waitlist control) [54] in both meta-analyses, such that the control group that received the standardized Web-based intervention without contact was included in the tailored Web-based intervention versus active control, and the waitlist control group was included in the tailored Web-based intervention versus standard care or waitlist control.

**Table 4:** Assessment tools and timing.

First author, year, reference	Pain (with score range)	Pain-related disability	Psychological well-being	Timing of posttreatment assessment		
				Short	Medium	Long
Andersson, 2003 [48]	0-5 NRS^a,b^	HDI^c^	HADS^d^	X		
Bossen, 2013 [49]	0-10 NRS	PASE^e^, KOOS/HOOS^f^	HADS	X		X
Buhrman, 2004 [50]	0-100 VAS^g^	MPI^h^	HADS	X	X	
Carpenter, 2012 [51]	0-10 NRS	RMDQ^i^	PCS^j^	X		
Chiauzzi, 2010 [52]	0-10 NRS	BPI^k^	PCS, DASS^l^	X	X	
Dear, 2013 [53]	0-10 NRS	RMDQ	PHQ-9^m^ (depression), GAD-7^n^ (anxiety)	X	X	
Dear, 2015 [54]	0-10 NRS	RMDQ	PHQ-9 (depression), GAD-7 (anxiety)	X	X	
Krein, 2013 [55]	0-10 NRS	RMDQ	PCS	X		
Kristjánsdóttir, 2013 [46,47]	0-100 VAS	FIQ^o^	PCS	X	X	X
Leveille, 2009 [56]	0-10 NRS	N/A^p^	N/A		X	
Lorig, 2006 [57]	0-10 VAS	IIS^q^, HAI^r^	N/A		X	X
Lorig, 2008 [58]	0-10 VAS	ALS^s^, HAQ^t^	N/A		X	X
Martorella, 2012 [62]	0-10 NRS	BPI	PCS	X		
Moessner, 2012 [59]	0-10 NRS	RMDQ	HADS	X	X	
Moessner, 2014 [39]	0-10 NRS	RMDQ	N/A	X	X	X
Shigaki, 2013 [60]	0-100 NRS with 5-point increment	AIMS^u^	CES-D^v^ (depression)	X		X
Ström, 2000 [61]	0-100 NRS	HDI	BDI^w^ (depression)	X		

^a^Headache index: means of noted pain intensity for each day summed, divided by the total number of registration days.

^b^NRS: numeric rating scale.

^c^HDI: Headache Disability Inventory.

^d^HADS: Hospital Anxiety and Depression Scale.

^e^PASE: Physical Activity Scale for the Elderly.

^f^KOOS/HOOS: Knee Osteoarthritis Outcome Score/Hip Injury Osteoarthritis Outcome Score.

^g^VAS: visual analog scale.

^h^MPI: Multidimensional Pain Inventory.

^i^RMDQ: Roland Morris Disability Questionnaire.

^j^PCS: Pain Catastrophizing Scale.

^k^BPI: Brief Pain Inventory.

^l^DASS: Depression Anxiety Stress Scale.

^m^PHQ-9: Patient Health Questionnaire.

^n^GAD-7: Generalized Anxiety Disorder.

^o^FIQ: Fibromyalgia Impact Questionnaire.

^p^N/A: not applicable.

^q^IIS: Illness Intrusiveness Rating Scale.

^r^HAI: Health Assessment Instrument.

^s^ALS: Activities Limitation Scale.

^t^HAQ: Health Assessment Questionnaire.

^u^AIMS: Arthritis Impact Measurement Scale.

^v^CES-D: Center for Epidemiologic Studies Depression Scale.

^w^BDI: Beck Depression Inventory.

We removed the control group that received the standardized Web-based intervention with optional contact from the meta-analysis, as it was impossible to evaluate the amount of contact with a therapist that was received. Overall, we included 16 studies in the 2 main meta-analyses: 11 in the tailored Web-based intervention versus standard care or waitlist control [39 49-51,53,55,57-61], and 4 in the tailored Web-based intervention versus active control [46-48,52,56]; we included 1 in both [54].

### Effects of Tailored Web-Based Interventions Versus Standard Care

#### Pain

**Figure 2:**
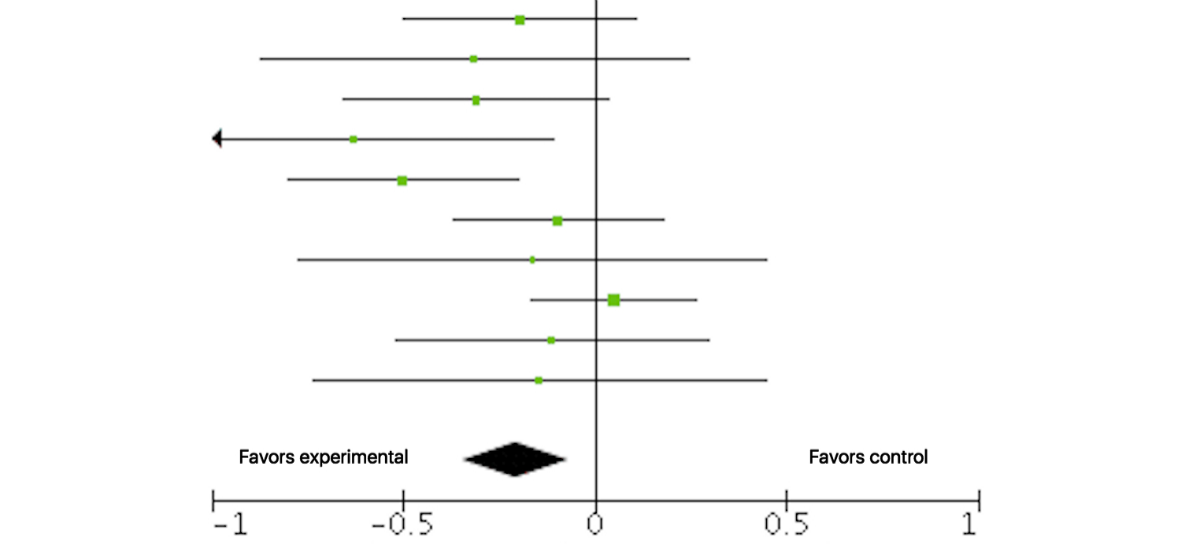
Forest plot of the standardized mean difference (95% CI) in pain intensity posttreatment between tailored Web-based interventions and standard care. Black diamond indicates overall treatment effect (tips=95% CI).

We entered 10 studies (n=1310) into an analysis of the short-term effect of Web-based tailored interventions on pain intensity. The overall effect of Web-based tailored interventions on pain intensity was beneficial, with a small effect size and no significant heterogeneity (SMD –0.21, 95% CI –0.34 to –0.0, *P*=.003; I^2^=29%). Figure 2 shows the forest plot of the SMD in pain intensity. The beneficial pain relief effect was not sustained at medium term (4 RCTs, n=987, SMD –0.08, 95% CI –0.30 to 0.13, *P*=.45; I^2^=48%) and long term (5 RCTs, n=1909, SMD –0.09, 95% CI –0.18 to 0.00, *P*=.05; I^2^=0%). Table 5 provides the details of analyses of the effect on pain intensity for each time point.

#### Pain-Related Disability

**Figure 3:**
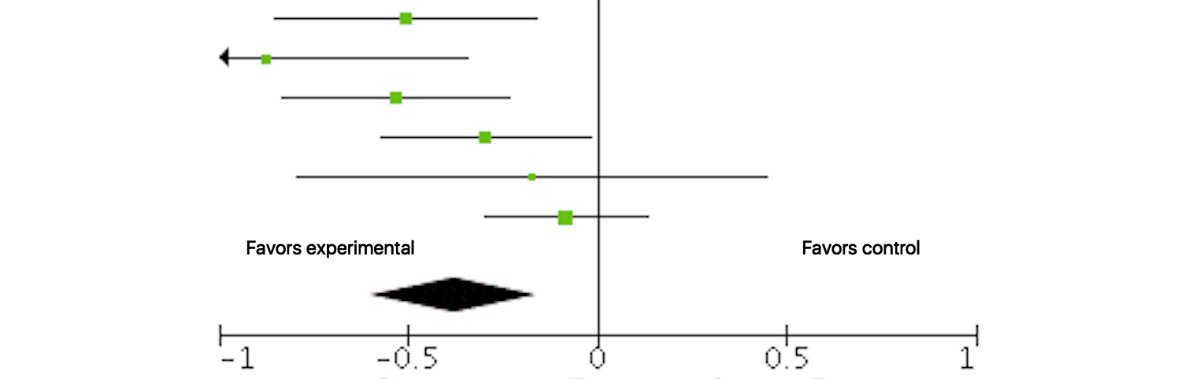
Forest plot of the standardized mean difference (95% CI) in pain-related disability posttreatment between tailored Web-based interventions and standard care. Black diamond indicates overall treatment effect (tips=95% CI).

We entered 6 studies (n=953) into the meta-analysis of the short-term effect of Web-based tailored interventions on pain-related disability. The overall effect was significantly beneficial with a small effect size, although heterogeneity was high (SMD –0.38, 95% CI –0.59 to –0.16, *P*<.001; I^2^=58%). Figure 3 shows the forest plot of the SMD in pain-related disability. We entered 3 studies (n=411) into the meta-analysis of the medium-term effect on pain-related disability. The overall effect, although in favor of the experimental group, was not significant (SMD –0.07, 95% CI –0.26 to 0.13, *P*=.49; I^2^=0%). The meta-analysis for long-term effect on pain-related disability could not be conducted due to the lack of assessments available at this time point.

#### Anxiety, Depression, and Pain Catastrophizing

We found no significant short-term reductions in anxiety (5 RCTs, n=507, SMD –0.08, 95% CI –0.50 to 0.34, *P*=.70; I^2^=79%) or depression (7 RCTs, n=635, SMD –0.33, 95% CI –0.66 to 0.00, *P*=.05; I^2^=73%) with the tailored Web-based intervention compared with standard care or waitlist control. Meta-analytic statistics could not be run for medium-term and long-term effects due to the lack of assessments available at these time points. Meta-analysis could also not be run for pain catastrophizing, which was measured in only 1 study. Multimedia Appendix 1 shows all other forest plots and SMD comparisons with standard care.

**Table 5:** Effect of tailored Web-based interventions on pain in the short, medium, and long terms compared with standard care.

Pain intensity		Study or subgroup				Weight (%)	Standard mean difference IV, random (95% CI)
		Experimental		Standard care			
		Mean (SD)	Total	Mean (SD)	Total		
**After completion of intervention^a^**							
	Bossen [49]	3.5 (4.93)	85	4.5 (5.28)	81	13.0	–0.20 (–0.50 to 0.11)
	Buhrman [50]	3.43 (1.68)	22	3.96 (1.63)	29	5.2	–0.32 (–0.87 to 0.24)
	Carpenter [51]	5.2 (1.5)	63	5.7 (1.7)	68	11.0	–0.31 (–0.65 to 0.04)
	Dear [53]	4.68 (1.7)	30	5.81 (1.85)	30	5.9	–0.63 (–1.15 to –0.11)
	Dear [54]	4.86 (1.79)	123	5.71 (1.5)	67	13.2	–0.50 (–0.80 to –0.20)
	Krein [55]	5.4 (2.2)	101	5.6 (2)	103	14.8	–0.09 (–0.37 to 0.18)
	Moessner [59]	2.17 (1.75)	18	2.54 (2.55)	24	4.4	–0.16 (–0.77 to 0.45)
	Moessner [39]	3.74 (2.09)	167	3.64 (2.03)	161	19.1	0.05 (–0.17 to 0.26)
	Shigaki [60]	3.68 (2.83)	44	4.02 (3.12)	49	8.7	–0.11 (–0.52 to 0.29)
	Strom [61]	2.29 (2.33)	20	2.6 (1.94)	25	4.7	–0.14 (–0.73 to 0.45)
	Total (95% CI)		673		637	100.0	–0.21 (–0.34 to –0.07)
**At follow-up (<6 months after completion of intervention)^b^**							
	Buhrman [50]	3.62 (2.04)	22	3.26 (2.16)	29	12.2	0.17 (–0.39 to 0.72)
	Lorig [58]	5.86 (2.44)	310	6.34 (2.31)	331	43.5	–0.20 (–0.36 to –0.05)
	Moessner [59]	2.67 (1.66)	26	3.46 (2.26)	24	12.0	–0.39 (–0.96 to 0.17)
	Moessner [39]	4.18 (2.24)	122	3.97 (2.23)	123	32.3	0.09 (–0.16 to 0.34)
	Total (95% CI)		480		507	100.0	–0.08 (–0.30 to 0.13)
**At follow-up (>6 months after completion of intervention)^c^**							
	Bossen [49]	3.5 (4.67)	76	3.8 (4.72)	71	7.7	–0.06 (–0.39 to 0.26)
	Lorig [57]	–0.37 (2.72)	354	–0.05 (2.46)	426	40.7	–0.12 (–0.26 to 0.02)
	Lorig [58]	5.77 (2.53)	307	6.1 (2.35)	344	34.1	–0.14 (–0.29 to 0.02)
	Moessner [39]	4.22 (2.32)	128	4.03 (2.54)	115	12.8	0.08 (–0.17 to 0.33)
	Shigaki [60]	4.14 (3.12)	43	3.92 (2.96)	45	4.6	0.07 (–0.35 to 0.49)
	Total (95% CI)		908		1001	100.0	–0.09 (–0.18 to 0.00)

^a^Heterogeneity: τ^2^=0.01; χ^2^_9_=12.7 (*P*=.18); I^2^=29%. Test for overall effect: Z=2.82 (*P*=.003).

^b^Heterogeneity: τ^2^=0.02; χ^2^_3_=5.8 (*P*=.12); I^2^=48%. Test for overall effect: Z=0.76 (*P*=.45).

^c^Heterogeneity: τ^2^=0.00; χ^2^_4_=2.9 (*P*=.58); I^2^=0%. Test for overall effect: Z=1.92 (*P*=.05).

### Effects of Tailored Web-Based Interventions Versus Active Control Group

#### Pain

The tailored Web-based intervention did not relieve pain significantly better than active control in the short term (4 RCTs, n=543, SMD –0.09, 95% CI –0.25 to 0.08, *P*=.32; I^2^=0%) or medium term (4 RCTs, n=630, SMD –0.14, 95% CI –0.29 to 0.02, *P*=.09; I^2^=0%). We could not analyze the long-term effects on pain because of lack of assessments available at this time point. Table 6 provides the details of analyses of the effect on pain intensity for each time point.

#### Pain-Related Disability

The tailored Web-based intervention did not relieve pain significantly better than the active control in the short term (2 RCTs, n=426, SMD –0.09, 95% CI –0.28 to 0.10, *P*=.37; I^2^=0%) or medium term (2 RCTs, n=411, SMD –0.01, 95% CI –0.20 to 0.19, *P*=.93; I^2^=0%). The long-term effect on pain-related disability could not be analyzed due to a lack of assessments available at this time point.

#### Anxiety, Depression, and Pain Catastrophizing

We entered 3 studies (n=450) into the meta-analysis of the short-term effect on anxiety. The overall effect was not significant (SMD –0.05, 95% CI –0.24 to 0.13, *P*=.56; I^2^=0%). In regard to medium-term effect on anxiety, we entered 2 studies (n=411) into the meta-analysis, and the overall effect was not significant (SMD 0.03, 95% CI –0.27 to 0.32, *P*=.87; I^2^=55%). Long-term effect on anxiety could not be explored due to a lack of assessments available at this time point.

We entered 3 studies (n=450) into the meta-analysis to analyze the short-term effect on depression. The overall effect was not significant (SMD –0.09, 95% CI –0.28 to 0.09, *P*=.33; I^2^=0%). We explored the medium-term effect on depression with 2 studies (n=411). The overall effect was not significant (SMD –0.04, 95% CI –0.30 to 0.21, *P*=.74; I^2^=43%). Long-term effects on depression could not be analyzed due again to a lack of assessments at this time point.

The only significant effect of the tailored Web-based intervention was observed for pain catastrophizing in the short term (2 RCTs, n=333, SMD –0.46, 95% CI –0.67 to –0.24, *P*<.001; I^2^=0%). Medium-term and long-term effects could not be explored due to lack of assessments available at this time point in this subgroup of studies. Multimedia Appendix 2 shows all forest plots and SMD comparisons with active control groups.

### Narrative Review

We could not include 1 study in the meta-analysis because it was the only study targeting acute pain after cardiac surgery [62]. The Web-based tailored intervention was compared with standard care. No effect on pain intensity was recorded. However, less pain interference with breathing and coughing (*P*=.04) was reported by the experimental group, as well as fewer negative pain beliefs and attitudes (*P*=.02).

### Funnel Plot Asymmetry and Possible Sources of Explanation

We examined funnel plot asymmetry for the meta-analysis of the effect on pain intensity when comparing tailored Web-based interventions versus standard care posttreatment (short term) solely because other analyses included too few studies (n<10) [64] (see Multimedia Appendix 3 for funnel plot).

**Table 6:** Effect of tailored Web-based interventions on pain in the short and medium terms compared with active control group.

Pain intensity		Study or subgroup				Weight (%)	Standard mean difference IV, random (95% CI)
		Experimental		Standard care			
		Mean (SD)	Total	Mean (SD)	Total		
**After completion of intervention^a^**							
	Andersson [48]	4 (4.9)	17	3.1 (2.4)	13	5.4	0.22 (–0.51 to 0.94)
	Chiauzzi [52]	5.13 (1.95)	95	5.35 (1.94)	104	36.8	–0.11 (–0.39 to 0.17)
	Dear [54]	4.86 (1.79)	123	5.2 (1.8)	104	41.7	–0.19 (–0.45 to 0.07)
	Kristjánsdóttir [46]	5.41 (2.41)	47	5.06 (2.34)	40	16.0	0.15 (–0.28 to 0.57)
	Total (95% CI)		282		261	100.0	–0.09 (–0.25 to 0.08)
**At follow-up (<6 months after completion of intervention)^b^**							
	Chiauzzi [52]	4.78 (2.44)	95	5.18 (2.24)	104	31.6	–0.17 (–0.45 to 0.11)
	Dear [54]	4.96 (2)	115	5.02 (1.93)	97	33.6	–0.03 (–0.30 to 0.24)
	Kristjánsdóttir [46]	5.19 (2.38)	37	5.85 (2.25)	40	12.2	–0.28 (–0.73 to 0.17)
	Leveille [56]	3.3 (2.9)	71	3.8 (3.1)	71	22.6	–0.17 (–0.50 to 0.16)
	Total (95% CI)		318		312	100.0	–0.14 (–0.29 to 0.02)

^a^Heterogeneity: τ^2^=0.00; χ^2^_3_=2.5 (*P*=.48); I^2^=0%. Test for overall effect: Z=0.99 (*P*=.32).

^b^Heterogeneity: τ^2^=0.00; χ^2^_3_=1.1 (*P*=.78); I^2^=0%. Test for overall effect: Z=1.70 (*P*=.09).

We observed some asymmetry at the bottom, possibly reflecting moderate heterogeneity due to the inclusion of small studies. Variations in samples coming from the general population as opposed to outpatients from a clinic could also be a potential explanation.

## Discussion

### Principal Findings

We explored the efficacy of tailored Web-based interventions for pain management in comparison with standard care or waiting-list controls and active controls. A total of 17 studies met the inclusion criteria. All studies used feedback as 1 of the tailoring mechanisms, and half of them (n=8) used a hybrid format including telephone or face-to-face contact with a therapist. Most of the studies compared the tailored Web-based intervention versus standard or waiting-list control (n=12). Only 1 study concerned acute pain, which we removed from the meta-analysis, resulting in 16 studies available for quantitative assessment. We evaluated 5 different outcomes: pain intensity (primary outcome), pain-related disability, anxiety, depression, and pain catastrophizing. We assessed effects according to 3 time intervals (short term: <1 month; medium term: 1-6 months; and long term: 6-12 months).

Compared with standard care or waiting list, pain intensity (10 RCTs, n=1310) and pain-related disability (6 RCTs, n=953) were improved immediately after the tailored Web-based intervention with small effect sizes (ie, <0.40). No other improvements were observed at follow-up in the medium and long terms. Other systematic reviews and meta-analyses on Web-based CBT [15,18,19], as well as traditional CBT [65], also reported small effects in the reduction of pain posttreatment compared with standard care or waiting list. One meta-analysis showed that small positive effects on pain-related disability were maintained at follow-up (3 months or more) [18]. However, evaluation of effects at follow-up did not distinguish between medium-term and long-term intervals (eg, 3 and 12 months). Moreover, the meta-analysis did not discriminate between active controls and standard or waitlist controls. Although results were in favor of tailored Web-based interventions, we found no statistically significant benefits for anxiety and depression in our study at any of the time points, and the level of heterogeneity was high. Another meta-analysis found a small effect on depression posttreatment compared with standard care. However, that analysis merged both depression and anxiety outcomes [19].

When comparing the active control group, we found no improvements for the primary outcome (pain intensity) or any of the outcomes except for a small effect size on pain catastrophizing (2 RCTs, n=333) immediately after the intervention. The 2 studies included in this analysis were quite different [46,52]. Although they both used content matching, they used different format (hybrid vs not), and a different approach (CBT and motivational vs CBT, acceptance and commitment therapy, and mindfulness). Nonetheless, it is important to underline that the levels of catastrophizing recorded were high. One meta-analysis on Web-based CBT found a small effect on pain catastrophizing posttreatment [19]. However, a high heterogeneity was reported and the comparator was standard care or waiting list. Our results in relation to the active control comparator are in concordance with a previous meta-analysis on traditional psychological therapies for chronic pain, which concluded that there was no evidence of efficacy of CBT and behavioral therapy on pain [65]. It is also noteworthy that meta-analyses conducted in our study with the active control group included very few studies.

Lastly, based on our results and previous results, a general observation can be made pertaining to the effects of Web-based interventions on chronic pain. Regardless of the type of comparison used (ie, usual treatment or active control), effects were redundantly small, which poses a question concerning the adequacy of pain reduction as an outcome. Previous authors [18] have suggested including participants with moderate to severe pain intensity at baseline in order to appreciate the benefits of these interventions. This was true for the only 2 studies in our review that reported moderate effects on pain [53,54]. All participants had a moderate level of pain intensity at baseline. Another avenue could be to focus on outcomes related to the concept of chronic pain acceptance and quality of life [66,67], as most interventions for chronic pain used a CBT approach aimed at reducing disability, depression, and anxiety, not necessarily reducing pain.

### Limitations and Future Research

Although our results are consistent with other meta-analyses, a major difference in our results is related to the heterogeneity of intervention approaches that we found in tailored Web-based interventions for pain management as opposed to traditional CBT. Selected studies included interventions profoundly influenced by CBT, but approaches were oftentimes multimodal and varied from wearing a pedometer and physical activity coaching to relaxation and mindfulness. Many of the interventions also included an educational aspect related to management of a specific chronic disease and wellness. The dosage of interventions was also very diverse, ranging from 2 sessions per week for 3 weeks [51] or 5 sessions per week for 4 weeks [46,47] to 12 to 15 weekly sessions [39] or even weekly reminders for 12 months [55]. This variation does not allow for gauging the influence of these interventions. Another observation regarding intervention content is that only half of the interventions used the mechanism of content matching, which is thought to be the essence of tailored approaches [21]. While the only 2 studies that reported moderate effects (ie, <0.50) on pain intensity did not use content matching but rather a hybrid format involving telephone contact with a therapist [53,54], carefully choosing behavioral change techniques that fit the targeted behavior could enhance the effects of these interventions [12,68]. The contribution of this tailoring mechanism is yet to be explored regarding pain management. When the number of studies available allows it, it would be interesting to examine effect according to tailoring mechanisms and dosage.

Attrition and fidelity of intervention delivery are challenges in Web-based interventions. The attrition rate ranged from 5% to 56% with an average of 22%, even though all interventions included some kind of feedback, with half of them using human interaction. This rate is similar to the rate found in another meta-analysis, underlining that there was no difference in attrition even when participants received more guidance as opposed to no reminders or feedback [19]. Questions remain regarding this issue and the efficacy of these methods. A meta-analysis on tailored Web-based interventions in general found that expert input does not necessarily mean more efficacy [30]. The intervention itself could also be one aspect of the problem. Indeed, interventions for chronic pain require engagement, and some involve frequent weekly activities and are of long duration. Web-based tailored interventions for chronic conditions should consider disease burden and the complexity of accomplishing certain tasks [30]. Although we cannot outline clear guidelines, among studies with higher dropout rates (<20%), interventions could include up to 5 sessions per week [46,47,58] or could last for 12 to 15 weeks [59]. Having a better understanding of which ingredient works for which patient and, consequently, using tailoring to adapt content could lead to more concise and efficient interventions. In fact, it has been observed that pain management interventions with a duration of less than 8 weeks are more effective [69]. Moreover, cost-benefit analyses would definitely be informative, as only small effects have been recorded.

In an effort to decrease heterogeneity in measures and their involved concepts when looking at pain-related disability, we focused on the 2 most commonly used measures: the Roland Morris Disability Questionnaire and the Brief Pain Inventory. This could have influenced our results. Although our study is 1 of the few making a distinction between comparators, we could include only 5 studies in the meta-analysis focusing on active control groups. Our results are in that sense to be interpreted with caution.

Lastly, we found only 1 intervention for acute pain, which highlights the need to explore the avenue of tailored Web-based interventions for this type of pain and for the prevention of chronic pain. The context and settings of acute pain are very different from the chronic spectrum and could lead to different outcomes. Hence, it has been shown that Web-based tailored interventions that are preventive in nature and targeting a general population, not a specific condition, were more successful [30]. Another possible avenue for intervention development is the consideration of sex differences. The majority of participants in the included studies were women, which was observed in other meta-analyses on Web-based interventions for pain management [18,19]. These interventions are tailored to some extent, but none of them have taken into account sex differences in terms of pain experience and coping styles. Although there is still some controversy around pain sensitivity differences, getting a better understanding of women’s needs as opposed to men’s needs could improve the uptake of interventions. A recent study (n=1371) in the context of a rehabilitation program found that women have better activity level, pain acceptance, and social support, while men report more fear of movement and mood disturbances [70].

### Implications and Conclusion

Although several meta-analyses and systematic reviews of Web-based interventions for pain management have been conducted, this is, to our knowledge, the first examining tailored Web-based interventions and using active control groups as a comparator. Tailored Web-based interventions did not prove to be more efficacious than standardized Web-based interventions in terms of pain intensity, pain-related disability, anxiety, and depression. Similar findings to other meta-analyses on Web-based interventions for pain management were generated, meaning that these interventions may have a short-term effect on pain intensity and disability compared with usual care. An interesting finding was that some efficacy was shown in pain catastrophizing compared with active control interventions.

Tailored Web-based interventions are a recent field of research among behavioral change interventions. Considering the diversity of approaches used in tailored Web-based interventions for chronic pain management, their efficacy is yet to be explored. Moreover, the scarcity of tailored Web-based interventions available for acute pain management reflects the multitude of possibilities for intervention development. Feedback was used in all studies but content matching, the most important tailoring ingredient, was used in only half of the studies. More studies would improve our understanding of the efficacy of these interventions, enabling subgroup analyses according to their therapeutic content and their level of tailoring (ie, content matching).

## Appendix

Multimedia Appendix 1 Forest plots and standardized mean differences: standard care.

Multimedia Appendix 2 Forest plots and standardized mean differences: active control.

Multimedia Appendix 3 Funnel plot: tailored Web-based interventions vs standard care (n=10).

## Abbreviations

CBT: cognitive behavioral therapy
NRS: numeric rating scale
RCT: randomized controlled trial
SMD: standardized mean difference
VAS: visual analog scale
