# Supplementary material for: Tailored Web-Based Interventions for Pain: Systematic Review and Meta-Analysis
Source: J Med Internet Res. 2017 Nov 10;19(11):e385. doi: 10.2196/jmir.8826 (PMC5701966; doi:10.2196/jmir.8826)

Forest plots and standardized mean differences

Tailored Web-based interventions vs. STANDARD CARE

1.1. Pain intensity after completion of intervention: short term

	Experimental			Standard Care				Std. Mean Difference
Study or Subgroup	Mean	SD	Total	Mean	SD	Total	Weight	IV, Random, 95% CI
Bossen 2013	3.5	4.93	85	4.5	5.28	81	13.0%	-0.20 [-0.50, 0.11]
Buhrman 2004	3.43	1.68	22	3.96	1.63	29	5.2%	-0.32 [-0.87, 0.24]
Carpenter 2012	5.2	1.5	63	5.7	1.7	68	11.0%	-0.31 [-0.65, 0.04]
Dear 2013	4.68	1.7	30	5.81	1.85	30	5.9%	-0.63 [-1.15, -0.11]
Dear 2015	4.86	1.79	123	5.71	1.5	67	13.2%	-0.50 [-0.80, -0.20]
Krein 2013	5.4	2.2	101	5.6	2	103	14.8%	-0.09 [-0.37, 0.18]
Moessner 2012	2.17	1.75	18	2.54	2.55	24	4.4%	-0.16 [-0.77, 0.45]
Moessner, 2013	3.74	2.09	167	3.64	2.03	161	19.1%	0.05 [-0.17, 0.26]
Shigaki 2013	3.68	2.83	44	4.02	3.12	49	8.7%	-0.11 [-0.52, 0.29]
Strom 2000	2.29	2.33	20	2.6	1.94	25	4.7%	-0.14 [-0.73, 0.45]
**Total (95% CI)**			**673**			**637**	**100.0%**	**-0.21 [-0.34, -0.07]**

Heterogeneity: $Tau^2 = 0.01$; $Chi^2 = 12.71$, df = 9 (P = 0.18); $I^2 = 29\%$
Test for overall effect: Z = 2.95 (P = 0.003)

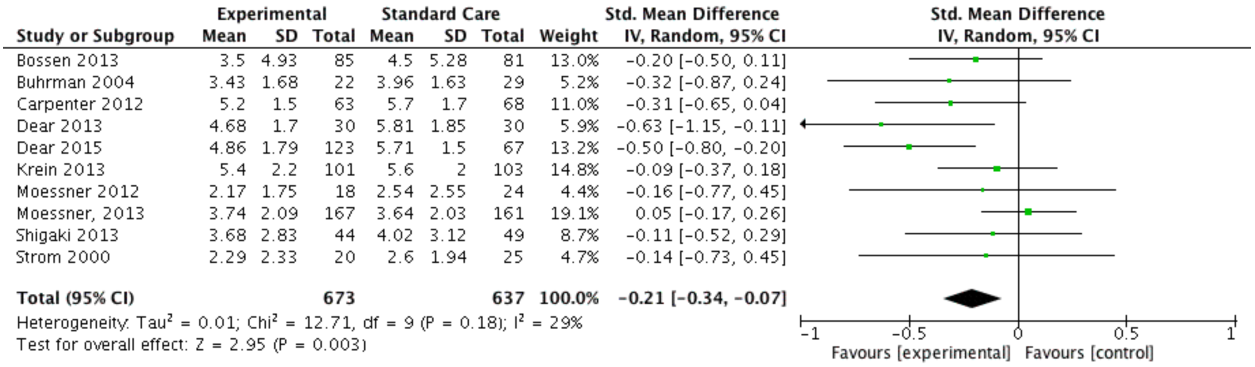

1.2. Pain intensity follow-up (<6 months after completion of intervention): medium term

	Experimental			Standard Care				Std. Mean Difference
Study or Subgroup	Mean	SD	Total	Mean	SD	Total	Weight	IV, Random, 95% CI
Buhrman 2004	3.62	2.04	22	3.26	2.16	29	12.2%	0.17 [-0.39, 0.72]
Lorig 2008	5.86	2.44	310	6.34	2.31	331	43.5%	-0.20 [-0.36, -0.05]
Moessner 2012	2.67	1.66	26	3.46	2.26	24	12.0%	-0.39 [-0.96, 0.17]
Moessner, 2013	4.18	2.24	122	3.97	2.23	123	32.3%	0.09 [-0.16, 0.34]
**Total (95% CI)**			**480**			**507**	**100.0%**	**-0.08 [-0.30, 0.13]**

Heterogeneity: $Tau^2 = 0.02$; $Chi^2 = 5.82$, df = 3 (P = 0.12); $I^2 = 48\%$
Test for overall effect: Z = 0.76 (P = 0.45)

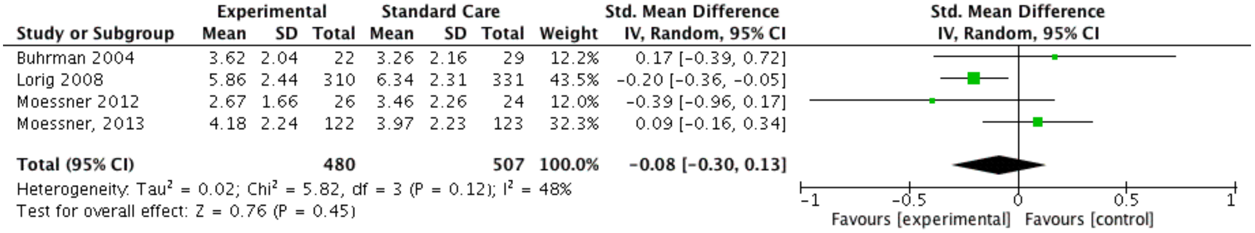

1.3. Pain intensity follow-up (>6 months after completion of intervention): long term

	Experimental			Standard Care				Std. Mean Difference
Study or Subgroup	Mean	SD	Total	Mean	SD	Total	Weight	IV, Random, 95% CI
Bossen 2013	3.5	4.67	76	3.8	4.72	71	7.7%	-0.06 [-0.39, 0.26]
Lorig 2006	-0.37	2.72	354	-0.05	2.46	426	40.7%	-0.12 [-0.26, 0.02]
Lorig 2008	5.77	2.53	307	6.1	2.35	344	34.1%	-0.14 [-0.29, 0.02]
Moessner, 2013	4.22	2.32	128	4.03	2.54	115	12.8%	0.08 [-0.17, 0.33]
Shigaki 2013	4.14	3.12	43	3.92	2.96	45	4.6%	0.07 [-0.35, 0.49]
**Total (95% CI)**			**908**			**1001**	**100.0%**	**-0.09 [-0.18, 0.00]**

Heterogeneity: $Tau^2 = 0.00$; $Chi^2 = 2.86$, df = 4 (P = 0.58); $I^2 = 0\%$
Test for overall effect: Z = 1.92 (P = 0.05)

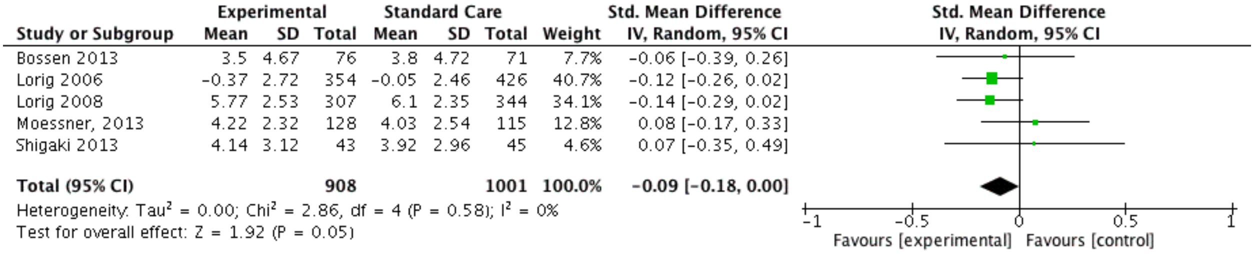

2.1. Pain related disability after completion of intervention: short term

Study or Subgroup	Experimental Mean	Experimental SD	Experimental Total	Standard Care Mean	Standard Care SD	Standard Care Total	Weight	Std. Mean Difference IV, Random, 95% CI
Carpenter 2012	13.5	5.8	63	16.3	5.2	68	17.1%	−0.51 [−0.85, −0.16]
Dear 2013	10.1	5.23	30	14.77	5.33	30	10.8%	−0.87 [−1.40, −0.34]
Dear 2015	11.05	5.63	123	13.97	5.17	67	19.2%	−0.53 [−0.83, −0.23]
Krein 2013	6.6	5.9	102	8.3	5.6	103	20.6%	−0.29 [−0.57, −0.02]
Moessner 2012	8.89	6.66	18	10.09	6.9	23	8.8%	−0.17 [−0.79, 0.44]
Moessner, 2013	11.4	5.67	167	11.87	5.68	159	23.5%	−0.08 [−0.30, 0.13]
**Total (95% CI)**			**503**			**450**	**100.0%**	**−0.38 [−0.59, −0.16]**

Heterogeneity: $Tau^2 = 0.04$; $Chi^2 = 11.97$, df = 5 (P = 0.04); $I^2 = 58\%$
Test for overall effect: Z = 3.43 (P = 0.0006)

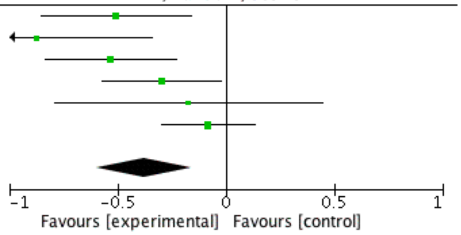

2.2. Pain related disability follow-up (<6 months after completion of intervention): medium term

Study or Subgroup	Experimental Mean	Experimental SD	Experimental Total	Standard Care Mean	Standard Care SD	Standard Care Total	Weight	Std. Mean Difference IV, Random, 95% CI
Carpenter 2012	11.9	5.9	63	12.9	6.9	55	28.6%	−0.16 [−0.52, 0.21]
Moessner 2012	9.42	5.7	26	9.96	6.47	23	11.9%	−0.09 [−0.65, 0.47]
Moessner, 2013	11.32	5.95	122	11.46	6.21	122	59.5%	−0.02 [−0.27, 0.23]
**Total (95% CI)**			**211**			**200**	**100.0%**	**−0.07 [−0.26, 0.13]**

Heterogeneity: $Tau^2 = 0.00$; $Chi^2 = 0.35$, df = 2 (P = 0.84); $I^2 = 0\%$
Test for overall effect: Z = 0.69 (P = 0.49)

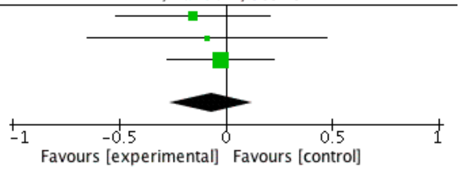

3. Anxiety after completion of intervention: short term

Study or Subgroup	Experimental Mean	Experimental SD	Experimental Total	Standard Care Mean	Standard Care SD	Standard Care Total	Weight	Std. Mean Difference IV, Random, 95% CI
Bossen 2013	3.5	4.7	85	4.2	4.76	79	23.2%	−0.15 [−0.45, 0.16]
Buhrman 2004	7.2	4	22	6	3.3	29	18.0%	0.33 [−0.23, 0.88]
Dear 2013	7.23	4.76	30	9.03	4.78	30	19.0%	−0.37 [−0.88, 0.14]
Dear 2015	4.91	4.4	123	7.89	5.29	67	23.3%	−0.63 [−0.93, −0.32]
Moessner 2012	10.06	2.01	18	8.75	1.84	24	16.5%	0.67 [0.04, 1.30]
**Total (95% CI)**			**278**			**229**	**100.0%**	**−0.08 [−0.50, 0.34]**

Heterogeneity: $Tau^2 = 0.17$; $Chi^2 = 18.82$, df = 4 (P = 0.0009); $I^2 = 79\%$
Test for overall effect: Z = 0.38 (P = 0.70)

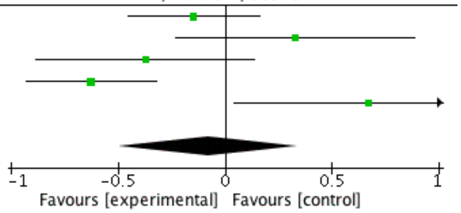

4. Depression after completion of intervention: short term

Study or Subgroup	Experimental Mean	Experimental SD	Experimental Total	Standard Care Mean	Standard Care SD	Standard Care Total	Weight	Std. Mean Difference IV, Random, 95% CI
Bossen 2013	2.6	5.17	85	3.2	4.96	78	17.4%	−0.12 [−0.43, 0.19]
Buhrman 2004	6	4.7	22	5.4	4	29	13.0%	0.14 [−0.42, 0.69]
Dear 2013	7.55	5.54	30	11.32	5.93	30	13.6%	−0.65 [−1.17, −0.13]
Dear 2015	6.3	4.57	123	11.11	5.51	67	17.3%	−0.97 [−1.29, −0.66]
Moessner 2012	5.5	4.01	18	6.21	3.67	24	12.0%	−0.18 [−0.79, 0.43]
Shigaki 2013	9.8	7.6	44	11.9	11.2	49	15.6%	−0.22 [−0.62, 0.19]
Strom 2000	6.93	7.41	14	7.86	4.85	22	11.1%	−0.15 [−0.82, 0.52]
**Total (95% CI)**			**336**			**299**	**100.0%**	**−0.33 [−0.66, −0.00]**

Heterogeneity: $Tau^2 = 0.14$; $Chi^2 = 22.35$, df = 6 (P = 0.001); $I^2 = 73\%$
Test for overall effect: Z = 1.98 (P = 0.05)

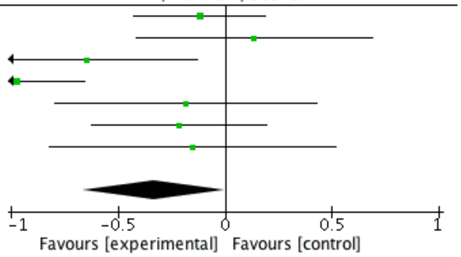

Supplement: Multimedia Appendix 1 [file jmir_v19i11e385_app1.pdf]
