# Supplementary material for: Tailored Web-Based Interventions for Pain: Systematic Review and Meta-Analysis
Source: J Med Internet Res. 2017 Nov 10;19(11):e385. doi: 10.2196/jmir.8826 (PMC5701966; doi:10.2196/jmir.8826)

Forest plots and standardized mean differences

Tailored Web-based interventions vs. ACTIVE CONTROL

1.1. Pain intensity after completion of intervention: short term

	Experimental			Active Control				Std. Mean Difference
Study or Subgroup	Mean	SD	Total	Mean	SD	Total	Weight	IV, Random, 95% CI
Andersson 2003	4	4.9	17	3.1	2.4	13	5.4%	0.22 [−0.51, 0.94]
Chiauzzi 2010	5.13	1.95	95	5.35	1.94	104	36.8%	−0.11 [−0.39, 0.17]
Dear 2015	4.86	1.79	123	5.2	1.8	104	41.7%	−0.19 [−0.45, 0.07]
Kristjansdottir 2013	5.41	2.41	47	5.06	2.34	40	16.0%	0.15 [−0.28, 0.57]
**Total (95% CI)**			**282**			**261**	**100.0%**	**−0.09 [−0.25, 0.08]**

Heterogeneity: Tau^2 = 0.00; Chi^2 = 2.46, df = 3 (P = 0.48); I^2 = 0%
Test for overall effect: Z = 0.99 (P = 0.32)

Std. Mean Difference
IV, Random, 95% CI

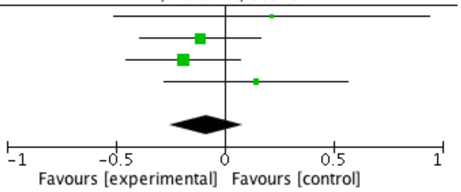

1.2. Pain intensity follow-up (<6 months after completion of intervention): medium term

	Experimental			Active Control				Std. Mean Difference
Study or Subgroup	Mean	SD	Total	Mean	SD	Total	Weight	IV, Random, 95% CI
Chiauzzi 2010	4.78	2.44	95	5.18	2.24	104	31.6%	−0.17 [−0.45, 0.11]
Dear 2015	4.96	2	115	5.02	1.93	97	33.6%	−0.03 [−0.30, 0.24]
Kristjansdottir 2013	5.19	2.38	37	5.85	2.25	40	12.2%	−0.28 [−0.73, 0.17]
Leveille 2009	3.3	2.9	71	3.8	3.1	71	22.6%	−0.17 [−0.50, 0.16]
**Total (95% CI)**			**318**			**312**	**100.0%**	**−0.14 [−0.29, 0.02]**

Heterogeneity: Tau^2 = 0.00; Chi^2 = 1.08, df = 3 (P = 0.78); I^2 = 0%
Test for overall effect: Z = 1.70 (P = 0.09)

Std. Mean Difference
IV, Random, 95% CI

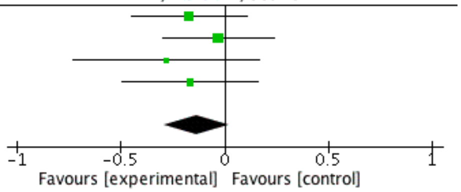

2.1. Pain related disability after completion of intervention: short term

	Experimental			Active Control				Std. Mean Difference
Study or Subgroup	Mean	SD	Total	Mean	SD	Total	Weight	IV, Random, 95% CI
Chiauzzi 2010	4.7	2.83	95	5.03	2.65	104	46.8%	−0.12 [−0.40, 0.16]
Dear 2015	11.05	5.63	123	11.36	5.22	104	53.2%	−0.06 [−0.32, 0.20]
**Total (95% CI)**			**218**			**208**	**100.0%**	**−0.09 [−0.28, 0.10]**

Heterogeneity: Tau^2 = 0.00; Chi^2 = 0.11, df = 1 (P = 0.74); I^2 = 0%
Test for overall effect: Z = 0.89 (P = 0.37)

Std. Mean Difference
IV, Random, 95% CI

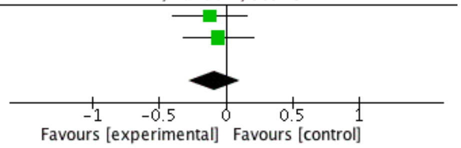

2.2. Pain related disability follow-up (<6 months after completion of intervention): medium term

	Experimental			Active Control				Std. Mean Difference
Study or Subgroup	Mean	SD	Total	Mean	SD	Total	Weight	IV, Random, 95% CI
Chiauzzi 2010	4.95	3.12	95	4.78	2.96	104	48.6%	0.06 [−0.22, 0.33]
Dear 2015	10.01	5.78	115	10.4	5.37	97	51.4%	−0.07 [−0.34, 0.20]
**Total (95% CI)**			**210**			**201**	**100.0%**	**−0.01 [−0.20, 0.19]**

Heterogeneity: Tau^2 = 0.00; Chi^2 = 0.40, df = 1 (P = 0.53); I^2 = 0%
Test for overall effect: Z = 0.09 (P = 0.93)

Std. Mean Difference
IV, Random, 95% CI

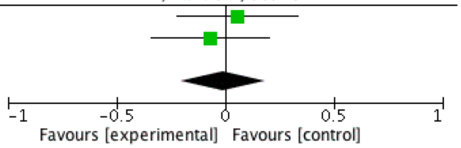

3.1. Anxiety after completion of intervention: short term

Study or Subgroup	Experimental Mean	Experimental SD	Experimental Total	Active Control Mean	Active Control SD	Active Control Total	Weight	Std. Mean Difference IV, Random, 95% CI
Andersson 2003	7.9	5.1	15	7	4.8	9	5.0%	0.17 [−0.65, 1.00]
Chiauzzi 2010	7.72	9.55	95	8.42	9.07	104	44.5%	−0.07 [−0.35, 0.20]
Dear 2015	4.91	4.4	123	5.16	3.91	104	50.5%	−0.06 [−0.32, 0.20]
**Total (95% CI)**			**233**			**217**	**100.0%**	**−0.05 [−0.24, 0.13]**

Heterogeneity: $Tau^2 = 0.00$; $Chi^2 = 0.31$, df = 2 (P = 0.85); $I^2 = 0\%$
Test for overall effect: Z = 0.58 (P = 0.56)

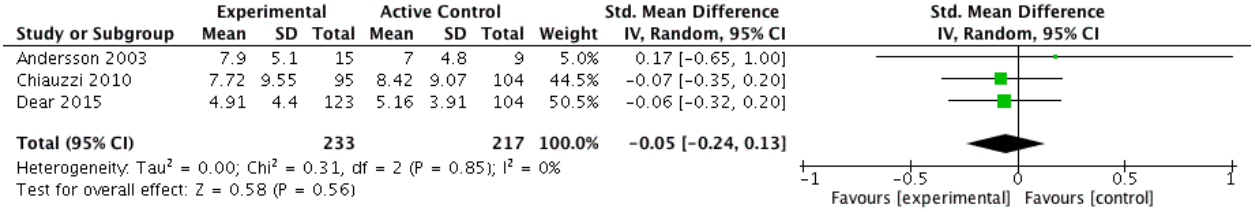

3.2. Anxiety follow-up (<6 months after completion of intervention): medium term

Study or Subgroup	Experimental Mean	Experimental SD	Experimental Total	Active Control Mean	Active Control SD	Active Control Total	Weight	Std. Mean Difference IV, Random, 95% CI
Chiauzzi 2010	7.22	8.97	95	8.32	8.57	104	49.4%	−0.13 [−0.40, 0.15]
Dear 2015	5.67	4.83	115	4.92	3.72	97	50.6%	0.17 [−0.10, 0.44]
**Total (95% CI)**			**210**			**201**	**100.0%**	**0.03 [−0.27, 0.32]**

Heterogeneity: $Tau^2 = 0.02$; $Chi^2 = 2.24$, df = 1 (P = 0.13); $I^2 = 55\%$
Test for overall effect: Z = 0.17 (P = 0.87)

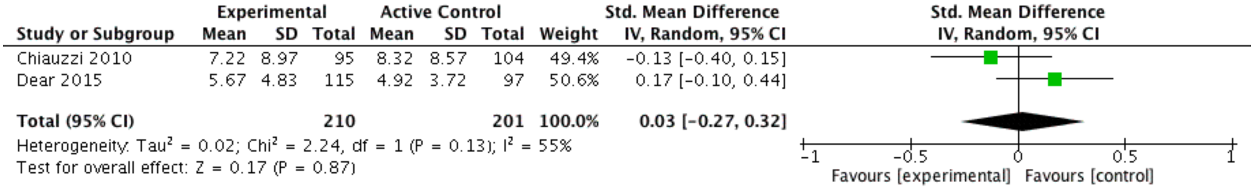

4.1. Depression after completion of intervention: short term

Study or Subgroup	Experimental Mean	Experimental SD	Experimental Total	Active Control Mean	Active Control SD	Active Control Total	Weight	Std. Mean Difference IV, Random, 95% CI
Andersson 2003	4.5	3.9	15	4.9	3.3	9	5.0%	−0.10 [−0.93, 0.72]
Chiauzzi 2010	11.15	10.52	95	11.44	9.99	104	44.5%	−0.03 [−0.31, 0.25]
Dear 2015	6.3	4.57	123	6.96	4.29	104	50.4%	−0.15 [−0.41, 0.11]
**Total (95% CI)**			**233**			**217**	**100.0%**	**−0.09 [−0.28, 0.09]**

Heterogeneity: $Tau^2 = 0.00$; $Chi^2 = 0.38$, df = 2 (P = 0.83); $I^2 = 0\%$
Test for overall effect: Z = 0.98 (P = 0.33)

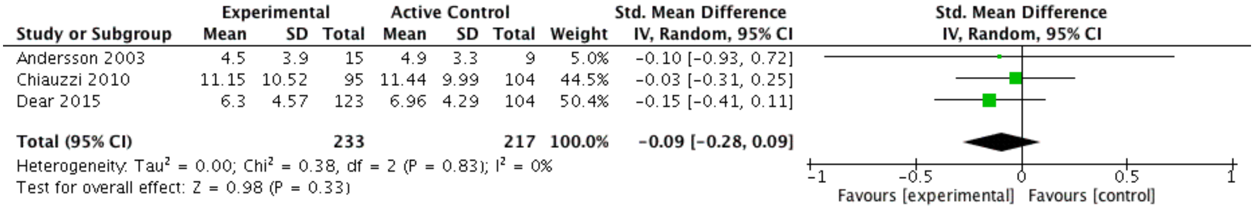

4.2. Depression follow-up (<6 months after completion of intervention): medium term

Study or Subgroup	Experimental Mean	Experimental SD	Experimental Total	Active Control Mean	Active Control SD	Active Control Total	Weight	Std. Mean Difference IV, Random, 95% CI
Chiauzzi 2010	10.55	12.18	95	12.65	11.42	104	49.1%	−0.18 [−0.46, 0.10]
Dear 2015	7.49	5.09	115	7.08	4.32	97	50.9%	0.09 [−0.18, 0.36]
**Total (95% CI)**			**210**			**201**	**100.0%**	**−0.04 [−0.30, 0.21]**

Heterogeneity: $Tau^2 = 0.02$; $Chi^2 = 1.77$, df = 1 (P = 0.18); $I^2 = 43\%$
Test for overall effect: Z = 0.33 (P = 0.74)

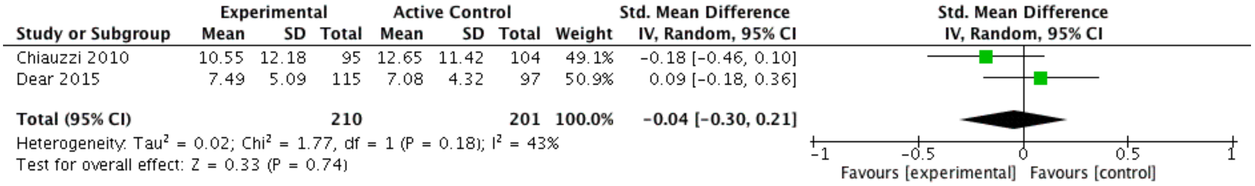

5. Pain catastrophizing after completion of intervention: short term

Study or Subgroup	Experimental Mean	Experimental SD	Experimental Total	Active Control Mean	Active Control SD	Active Control Total	Weight	Std. Mean Difference IV, Random, 95% CI
Chiauzzi 2010	14.92	12.67	95	21.08	12.14	104	59.5%	−0.50 [−0.78, −0.21]
Kristjansdottir 2013	12.32	9.22	69	16.07	9.48	65	40.5%	−0.40 [−0.74, −0.06]
**Total (95% CI)**			**164**			**169**	**100.0%**	**−0.46 [−0.67, −0.24]**

Heterogeneity: $Tau^2 = 0.00$; $Chi^2 = 0.18$, df = 1 (P = 0.67); $I^2 = 0\%$
Test for overall effect: Z = 4.10 (P < 0.0001)

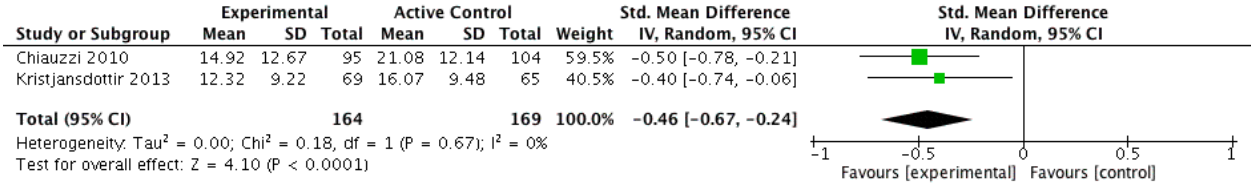

Supplement: Multimedia Appendix 2 [file jmir_v19i11e385_app2.pdf]
