# Supplementary figures and images for: Tailored Web-Based Interventions for Pain: Systematic Review and Meta-Analysis
Source: J Med Internet Res. 2017 Nov 10;19(11):e385. doi: 10.2196/jmir.8826 (PMC5701966; doi:10.2196/jmir.8826)

Funnel plot: Tailored Web-based interventions vs Standard care (n=10)

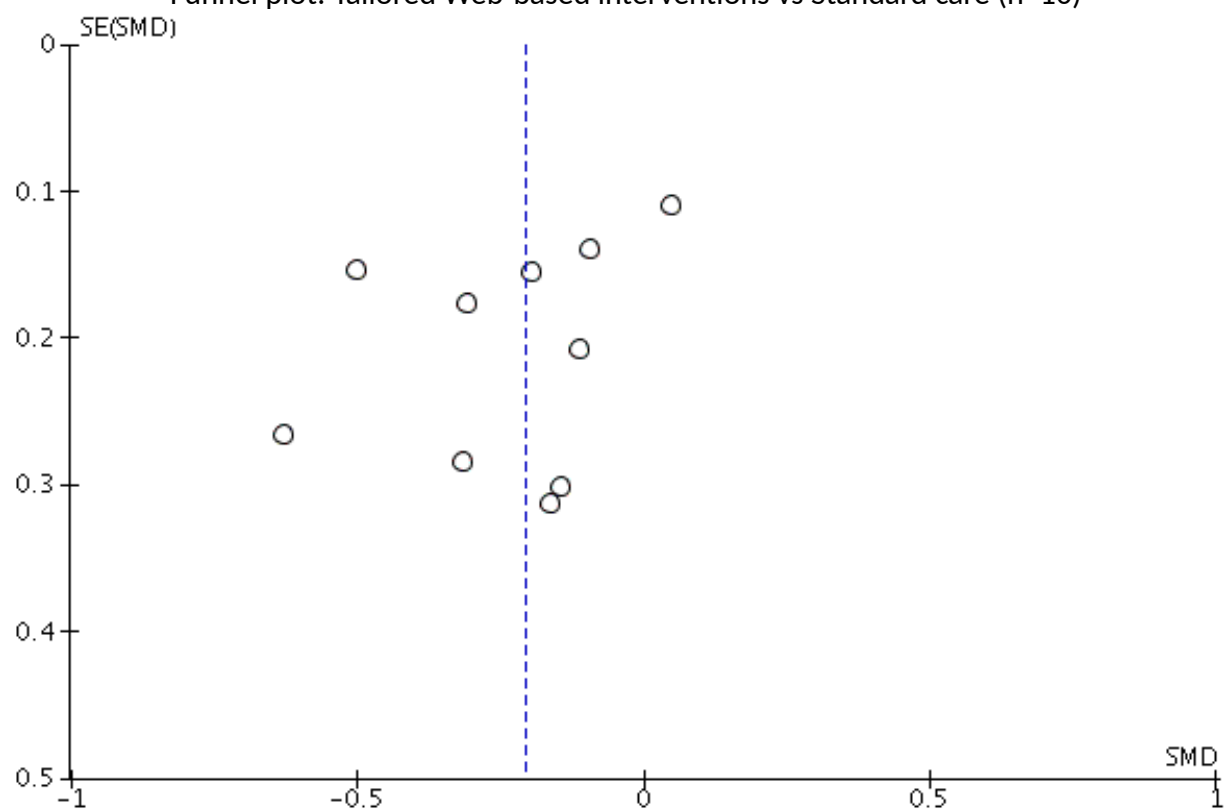

Supplement: Multimedia Appendix 3 [file jmir_v19i11e385_app3.pdf]
